# Machine learning-driven model for predicting knowledge, attitudes, and practices regarding medication safety among residents in Hubei, China

**DOI:** 10.3389/fpubh.2025.1574531

**Published:** 2025-06-02

**Authors:** Chao Mei, San-Lan Wu, Tao Zhou, Yong-Ning Lv, Yu Zhang, Chen Shi, Wei-Jing Gong

**Affiliations:** ^1^Department of Pharmacy, Union Hospital, Tongji Medical College, Huazhong University of Science and Technology, Wuhan, China; ^2^Hubei Province Clinical Research Center for Precision Medicine for Critical Illness, Wuhan, China

**Keywords:** KAP, rational drug use, machine learning, medication behavior, model

## Abstract

**Objective:**

To evaluate the current state and determinants of medication safety knowledge, attitudes, and practices (KAP) among residents in Hubei Province, and to offer guidance for targeted educational initiatives.

**Methods:**

A standardized questionnaire from the Science and Technology Development Center of the Chinese Pharmaceutical Association was utilized. Responses were scored systematically. Univariate and multivariate Logistic regression analyses, along with machine learning (ML) techniques, were applied to identify risk factors associated with medication safety KAP.

**Results:**

Out of 1,065 distributed questionnaires, 1,042 were valid (91.8% response rate). The study revealed that 30.2% of residents demonstrated 'excellent' medication knowledge, while attitude and practice scores were lower 10.3 and 46.3%, respectively. Univariate analysis indicated that age, monthly income, employment status, and occupation significantly influenced KAP. Multivariate analysis further identified age (≥65 years: OR = 0.27), education level (Middle school: OR = 0.36, Primary school: OR = 0.16), occupation (Healthcare workers: OR = 3.67), and medical insurance coverage (Basic social medical insurance: OR = 17.48, Out-of-pocket medical care: OR = 7.44, Publicly-funded medical care: OR = 11.92) as independent risk factors affecting the total KAP score. In evaluating ML models for predicting KAP, the eXtreme Gradient Boosting (XGB) model showed the best performance for predicting knowledge (training accuracy: 0.7014, Kappa: 0.3045; validation accuracy: 0.6186, Kappa: 0.1004). The Fully Connected Neural Network (FCNN) was optimal for attitude prediction (training accuracy: 0.7205, Kappa: 0.0778; validation accuracy: 0.7019, Kappa: 0.0008). The Ordered Multinomial Logistic Regression model was most accurate for practice prediction (training accuracy: 0.6471, Kappa: 0.3421; validation accuracy: 0.6302, Kappa: 0.3153). And the Deep Neural Network (DNN) model demonstrated the highest accuracy for predicting the total score (training accuracy: 0.7387, Kappa: 0.3211; validation accuracy: 0.7074, Kappa: 0.1902).

**Conclusion:**

Residents of Hubei have a fundamental grasp of medication safety but also harbor certain misconceptions. Effective pharmaceutical science communication should take into account the characteristics of the residents and the identified risk factors.

## 1 Introduction

As medical paradigms shift and public self-care awareness grows, self-medication has become a prevalent and convenient method of health management worldwide (1–3). While self-medication can enhance health and reduce escalating healthcare costs (4), it also poses significant risks and safety concerns due to the widespread lack of professional pharmaceutical knowledge, insufficient understanding of drug information, and poor medication adherence among the general population (5–7). The risks associated with self-medication include self-misdiagnosis, inappropriate drug selection, non-adherence to medication instructions, irrational drug use, ignorance of contraindications, and disregard for physiological differences in medication (8, 9). These errors can worsen illnesses, cause drug-induced injuries, and even lead to drug abuse, posing a serious threat to public health.

Statistics reveal that medication errors result in billions of dollars in losses each year. In response, the World Health Organization (WHO) has launched the Global Patient Safety Challenge titled ‘Medication Without Harm’, which aims to raise public awareness about medication safety (10). In China, irrational drug use accounts for 11 to 26% of total drug prescriptions, leading to the hospitalization of at least 2.5 million patients annually due to adverse drug reactions and approximately 190,000 deaths from improper medication use (11). Hubei Province, with its high population density, faces a significant challenge in ensuring medication safety among its residents, which poses a severe risk to their health. Therefore, understanding the knowledge, attitudes, and practices (KAP) of residents regarding medication is crucial, as is developing rational intervention measures and enhancing their awareness of safe medication practices (12–14).

Recent advancements in artificial intelligence (AI) and machine learning (ML) have revolutionized drug safety assessments. ML models, such as eXtreme Gradient Boosting (XGBoost) and Deep Neural Networks (DNNs), enable predictive analytics for adverse drug reactions and personalized risk stratification (15, 16). These technologies address limitations in traditional statistical methods by handling complex, high-dimensional datasets and identifying non-linear relationships between variables (17). However, limited studies apply these techniques to evaluate population-level medication KAP.

This study aims to conduct a questionnaire survey to investigate the KAP of medication among permanent residents in Hubei Province, providing a comprehensive understanding of the current medication situation and associated risk factors. By employing multivariate logistic regression analysis and ML techniques, we aim to establish accurate prediction models to forecast the risks associated with residents’ self-medication. This research will shed light on the state of medication safety and the challenges faced in Hubei Province, offering a scientific foundation for the development of rational drug use strategies and informing targeted pharmaceutical science education and outreach efforts.

## 2 Methods

### 2.1 Subjects

This survey conducted a comprehensive analysis of the disparities in KAP regarding safe medication use among residents, considering various factors such as age, gender, occupation, education level, and more. Utilizing a non-probability sampling technique, the questionnaires were distributed from August to November 2024 to permanent residents across Hubei Province, encompassing cities like Wuhan, Ezhou, Yichang, Jingzhou, Huanggang, Enshi, Huangshi, Xiaogan, Suizhou, Shiyan, and Jingmen. The criteria for participant inclusion were as follows: (1) individuals aged 18 or above, irrespective of gender; (2) respondents who were conscious, mentally healthy, and without significant cognitive impairments; (3) those who voluntarily agreed to participate after being informed about the survey’s objectives and significance by the interviewers. Participants were excluded if they had language, hearing, cognitive, or other impairments that would impede their cooperation with the survey. This study was approved by the Ethics Committee of the Union Hospital, Tongji Medical College, Huazhong University of Science and Technology ([2024] shen (0589–01)).

### 2.2 Questionnaire and quality control

The survey was conducted using an online questionnaire form accessible at this link^1^. Before initiating the survey, all surveyors participated in a thorough training program. This program included comprehensive coverage of the questionnaire’s content, communication strategies, potential challenges that might be encountered during the survey, and effective solutions to these challenges. The training aimed to equip each surveyor with the ability to clearly articulate the survey’s objectives and significance to participants and to provide consistent responses to any questions that arose without bias, ensuring the respondents’ decisions remained uninfluenced.

After securing the consent of the respondents, they were guided to complete the questionnaire anonymously and honestly. For those who required assistance in completing the questionnaire, surveyors were on hand to provide support. To maintain the integrity of the data, the system was configured to allow only one submission per IP address. Additionally, any questionnaires completed in less than 100 s were discarded to eliminate responses that were either careless or insincere. Questionnaires with identical answers to all questions were also considered invalid to prevent any form of response manipulation.

### 2.3 Evaluation criteria

Beyond gathering basic demographic data, researchers assigned positive or negative values to the responses of 63 questions, categorizing them based on their alignment with safe medication practices among residents. A higher aggregate score indicated a more profound level of KAP regarding medication safety among the participants. Concurrently, a score rate was calculated using the formula: ‘Score Rate = (Score / Total Possible Score) × 100%’. This score rate was then used to classify the participants’ performance into three tiers: ‘excellent’ for a score rate of 80% or above, ‘adequate’ for a score rate between 60 and 79%, and ‘poor’ for a score rate below 60% (15, 16).

### 2.4 Statistical analysis

Given the non-normal distribution of KAP scores, this study employed the median (M) and interquartile range (P25, P75) for data representation. For statistical analysis, non-parametric tests were selected: the Mann–Whitney U test was utilized for comparisons between two groups, while the Kruskal-Wallis test was applied for assessments across multiple groups. Additionally, both univariate and multivariate logistic regression analyses were performed.

To develop predictive models for KAP and overall scores, seven ML approaches were employed: Ordered Multinomial Logistic Regression, Random Forest, XGBoost, Support Vector Machine (SVM), DNN, Fully Connected Neural Network (FCNN), and K-Nearest Neighbors (KNN). The methodology involved encoding categorical independent variables using one-hot encoding, with the dependent variable being an ordered categorical variable. The dataset was randomly stratified into a training set (70%) and a validation set (30%). Variable selection was performed using the Least Absolute Shrinkage and Selection Operator and BORUTA algorithms.

To ensure the reliability of our experimental results, a 10-fold cross-validation technique was implemented. Furthermore, key performance indicators, including accuracy, precision, recall, and the F1-score, were calculated using confusion matrices to comprehensively evaluate the models’ effectiveness. All analyses presented in this paper were conducted using R version 4.4.2 and Python 3.10.0.

## 3 Results

### 3.1 General information of participants

A total of 1,065 online questionnaires were collected, yielding 1,042 valid responses, which corresponds to an effective response rate of 97.93%. Among the respondents, 37.60% were male and 62.96% were female. The majority of the respondents, 94.53%, were aged between 19 and 64 years old. Residents aged ≥65 had significantly lower practice scores (median = 94.0 vs. 112.0 in 19–34 years, *p* < 0.01). Regarding monthly income, the most prevalent groups were those earning 2,000–4,000 yuan (17.37%), 4,000–6,000 yuan (22.36%), and above 6,000 yuan (38.29%). Urban dwellers constituted 77.06% of the sample, while rural residents made up 22.94%. Notably, a majority of the respondents, 66.32%, held a bachelor’s degree or higher (Table 1). For medication knowledge, 30.2% of residents demonstrated excellence, 62.4% adequacy, and 7.4% poor knowledge. Regarding medication attitude, 10.3% showed excellence, 19% adequacy, and 70.7% exhibited a poor attitude. In medication practice, 46.3% of residents had excellent practice, 47.7% adequacy, and 6% poor practice.

**Table 1:** Score distribution of KAP survey of residents in Hubei province.

Characteristics		*N*	Percentage (%)	Knowledge (Q37-60)		Practice (Q9-36)		Attitude (Q61-71)	
				*M* (*P*_25_, *P*_75_)	*p*	*M* (*P*_25_, *P*_75_)	*p*	*M* (*P*_25_, *P*_75_)	*p*
Sex	Male	386	37.04%	89.0 (80.0, 97.0)	0.01	109.5 (98.0, 121.0)	0.08	30.0 (26.0. 34.0)	0.16
	Female	656	62.96%	91.0 (85.0, 98.0)		111.0 (101.0, 122.0)		29.0 (26.0, 33.0)	
Age	19–34	421	40.40%	90.0 (83.0, 97.0)	<0.01	112.0 (105.0, 124.0)	<0.01	30.0 (27.0, 35.0)	0.12
	35–49	386	37.04%	92.0 (86.0, 99.0)		111.0 (103.0, 124.25)		28.5 (26.0, 33.0)	
	50–64	178	17.08%	91.0 (79.0, 97.0)		106.0 (93.0, 112.0)		28.5 (26.0, 32.25)	
	≥65	57	5.47%	87.0 (72.0, 93.5)		94.0 (84.0, 104.0)		29.0 (26.0, 32.5)	
Monthly income	<1,000	138	13.24%	87.0 (77.0, 93.25)	<0.01	95.0 (109.0, 117.0)	<0.01	27.0 (31.0, 36.0)	0.19
	1,000–2000	91	8.73%	79.0 (87.0, 92.0)		95.0 (106.0, 115.0)		27.0 (30.0, 34.0)	
	2000–4,000	181	17.37%	86.0 (91.0, 98.0)		99.0 (108.0, 119.5)		26.0 (28.0, 32.0)	
	4,000–6,000	233	22.36%	83.5 (91.0, 98.5)		100.0 (110.0, 121.0)		26.0 (29.0, 33.0)	
	>6,000	399	38.29%	85.0 (92.0, 99.0)		106.0 (112.0, 126.0)		26.0 (29.0, 34.0)	
Place of residence	Urban areas	803	77.06%	91.0 (85.0, 98.0)	<0.01	102.0 (111.0, 124.0)	<0.01	26.0 (29.0, 34.0)	0.22
	Rural areas	239	22.94%	79.0 (89.0, 95.0)		92.0 (108.0, 116.0)		27.0 (30.0, 34.0)	
Basic social medical insurance	Have	846	81.19%	92.0 (86.0, 98.0)	<0.01	111.0 (104.0, 123.0)	<0.01	29.0 (26.0, 33.0)	0.03
	Do not have	196	18.81%	84.5 (73.0, 93.0)		99.5 (84.25, 115.0)		30.0 (27.0, 35.0)	
Commercial insurance	Have	174	16.70%	90.0 (83.0, 98.0)	0.73	111.0 (101.75, 122.25)	0.36	29.0 (26.0, 35.25)	0.67
	Do not have	868	83.30%	91.0 (83.0, 97.0)		110.0 (100.0, 122.0)		29.0 (26.0, 34.0)	
Out-of-pocket medical care	Have	62	5.95%	81.5 (71.75, 92.0)	<0.01	98.0 (83.0, 110.25)	<0.01	30.0 (27.0, 38.0)	0.26
	Do not have	980	94.05%	91.0 (84.0, 98.0)		111.0 (101.0, 122.0)		29.0 (26.0, 34.0)	
Publicly-funded medical care	Have	76	7.29%	89.5 (76.25, 97.75)	0.10	107.5 (95.25, 122.75)	0.05	31.0 (27.0, 35.75)	0.02
	Do not have	966	92.71%	91.0 (84.0, 98.0)		110.0 (101.0, 122.0)		29.0 (26.0, 34.0)	
Other medical security	Have	38	3.65%	91.0 (84.0, 98.0)	<0.01	110.0 (101.0, 122.0)	0.01	29.0 (26.0, 34.0)	0.69
	Do not have	1,004	96.35%	84.0 (72.0, 87.25)		96.5 (87.75, 113.5)		29.5 (27.0, 34.0)	
Education level	Graduate student	281	26.97%	91.0 (84.0, 99.0)	<0.01	115.0 (107.5, 128.0)	<0.01	30.0 (27.0, 34.0)	0.03
	Bachelor	410	39.35%	91.5 (85.0, 98.0)		112.0 (106.0, 125.0)		29.0 (26.0, 34.0)	
	Junior college	135	12.96%	91.0 (83.0, 98.0)		107.0 (100.0, 115.0)		27.0 (26.0, 31.0)	
	Technical secondary or high school	103	9.88%	90.0 (84.0, 97.0)		104.0 (94.0, 112.0)		28.0 (26.0, 32.0)	
	Middle school	70	6.72%	90.5 (78.0, 95.0)		101.5 (85.0, 108.0)		29.0 (26.0, 32.0)	
	Primary school	43	4.13%	77.0 (71.0, 90)		84.0 (81.0, 96.0)		30.0 (27.0, 36.0)	
Employment	Currently employed	719	69.00%	92.0 (85.0, 99.0)	<0.01	112.0 (105.0, 125.0)	<0.01	29.0 (26.0, 34.0)	0.10
	Retired	113	10.84%	90.0 (80.0, 96.0)		101.0 (91.0, 110)		29.0 (26.0, 31.0)	
	Unemployed or jobless	210	20.15%	87.0 (78.0, 94.0)		108.0 (94.0, 117.0)		30.0 (27.0, 34.0)	
Occupation	Factory workers	107	10.27%	90.0 (83.0, 96.0)	<0.01	107.0 (100.0, 115.0)	<0.01	28.0 (26.0, 33.0)	0.00
	Company employees	178	17.08%	90.5 (84.0, 96.0)		108.0 (99.0, 117.0)		28.0 (27.0, 32.0)	
	Government cadres	55	5.28%	90.0 (82.0, 96.0)		107.0 (95.0, 118.0)		29.0 (26.0, 33.0)	
	Healthcare workers	264	25.34%	95.0 (88.0, 105.0)		120.5 (111.0, 132.0)		30.0 (26.0, 36.75)	
	Teachers	87	8.35%	90.0 (80.0, 98.0)		106.0 (95.0, 122.0)		29.0 (26.0, 35.0)	
	Business managers	26	2.50%	89.5 (72.5, 97.0)		106.5 (86.25, 121.25)		32.5 (28.75, 38.0)	
	Freelancers	73	7.01%	86.0 (78.0, 93.5)		104.0 (90.5, 112.0)		28.0 (26.5, 32.0)	
	Students	127	12.19%	88.0 (81.0, 95.0)		113.0 (106.0, 123.0)		31.0 (27.0, 35.0)	
	Others	125	0.119961612	90.0 (84.5, 97.5)		105.0 (94.0, 112.0)		28.0 (26.0, 32.0)	

*N*., number; Q37-60, question 37–60; M (P25, P75), median (percentage 25, percentage 75); Q9-36, question 9-36; Q61-71, question 61-71.

### 3.2 Influencing factors on medication KAP among Hubei residents

Univariate analysis indicated that age, monthly income, medical insurance, place of residence, education level, and occupation were significant predictors for knowledge, practice, and total scores. Specifically, females showed 1.42-fold higher odds of superior knowledge compared to males (95% CI: 1.09–1.84, *p* < 0.01). Individuals aged ≥65 years exhibited significantly reduced likelihoods across all metrics compared to the 19–34 reference group (knowledge: OR = 0.42, 95% CI: 0.23–0.77; practice: OR = 0.12, 95% CI: 0.07–0.22; total score: OR = 0.15, 95% CI: 0.08–0.28; all *p* < 0.01). Higher monthly income (≥6,000 vs. <1,000) was associated with elevated odds ratios (knowledge: OR = 2.76, 95% CI: 1.82–4.20; practice: OR = 1.80, 95% CI: 1.24–2.63; total score: OR = 2.86, 95% CI: 1.84–4.45; all *p* < 0.01). Urban residents showed lower odds relative to rural counterparts (knowledge: OR = 0.59, 95% CI: 0.43–0.80; practice: OR = 0.55, 95% CI: 0.41–0.73; total score: OR = 0.48, 95% CI: 0.35–0.67; all *p* < 0.01). Basic social medical insurance coverage significantly increased odds (knowledge: OR = 3.21, 95% CI: 2.25–4.57; practice: OR = 3.34, 95% CI: 2.40–4.66; total score: OR = 3.97, 95% CI: 2.69–5.88; all *p* < 0.01), whereas reliance on out-of-pocket payments decreased odds across all domains (knowledge: OR = 0.28, 95% CI: 0.16–0.50; practice: OR = 0.22, 95% CI: 0.13–0.38; total score: OR = 0.23, 95% CI: 0.13–0.42; all *p* < 0.01).

For knowledge scores, independent predictors included monthly income (≥6,000 vs. <1,000: OR = 2.41, 95% CI: 1.32–4.40, *p* < 0.01), education level (primary school vs. graduate: OR = 0.39, 95% CI: 0.16–0.94, *p* < 0.01), occupation (healthcare vs. factory workers: OR = 2.86, 95% CI: 1.70–4.82, *p* < 0.01), basic social medical insurance (OR = 6.42, 95% CI: 2.27–18.16, *p* < 0.01), and publicly-funded medical coverage (OR = 4.30, 95% CI: 1.37–13.49, *p* < 0.01). For practice and total scores, independent risk factors included age (≥65 vs. 19–34: practice OR = 0.29, 95% CI: 0.13–0.60, *p* < 0.01; total OR = 0.27, 95% CI: 0.12–0.61, *p* < 0.01), education level (primary school vs. graduate student: practice OR = 0.10, 95% CI: 0.04–0.24, *p* < 0.01; total OR = 0.16, 95% CI: 0.06–0.39, p < 0.01), occupation (healthcare workers vs. factory workers: practice OR = 3.62, 95% CI: 2.17–6.04, *p* < 0.01; total OR = 3.67, 95% CI: 2.08–6.48, *p* < 0.01), basic social medical insurance (have vs. do not have: practice OR = 10.05, 95% CI: 3.47–29.09, *p* < 0.01; total OR = 17.48, 95% CI: 5.94–51.41, *p* < 0.01), out-of-pocket medical care (have vs. do not have: practice OR = 3.47, 95% CI: 1.07–11.32, *p* = 0.04; total score OR = 7.44, 95% CI: 2.19–25.29, *p* < 0.01), and other medical security forms (have vs. do not have: practice OR = 4.35, 95% CI: 1.24–15.22, *p* = 0.02; total OR = 5.54, 95% CI: 1.48–20.73, *p* = 0.01). Notably, no factor was found to significantly impact the attitude score alone in the multivariate analysis (Table 2).

**Table 2:** Univariable and multivariable logistic analysis of risks factors influencing KAP scores.

	Univariate analysis		Multivariate analysis			Univariate analysis		Multivariate analysis	
Characteristics	OR (95%CI)	*p*	OR (95%CI)	*p*	Characteristics	OR (95%CI)	*p*	OR (95%CI)	*p*
Knowledge					Attitude				
Sex					Sex				
Male	1.00 (Reference)		1.00 (Reference)		Male	1.00 (Reference)		1.00 (Reference)	
Female	1.42 (1.09 ~ 1.84)	<0.01	1.26 (0.96 ~ 1.66)	0.10	Female	0.82 (0.63 ~ 1.08)		0.86 (0.65 ~ 1.14)	0.30
Age					Age				
19–34	1.00 (Reference)		1.00 (Reference)		19–34	1.00 (Reference)		1.00 (Reference)	
35–49	1.27 (0.96 ~ 1.68)	0.09	1.28 (0.92 ~ 1.79)	0.14	35–49	0.76 (0.56 ~ 1.02)	0.07	0.78 (0.55 ~ 1.10)	0.16
50–64	1.03 (0.72 ~ 1.47)	0.89	1.35 (0.84 ~ 2.17)	0.22	50–64	0.64 (0.43 ~ 0.94)	0.02	0.72 (0.44 ~ 1.17)	0.19
≥65	0.42 (0.23 ~ 0.77)	<0.01	0.50 (0.24 ~ 1.05)	0.07	≥65	0.59 (0.32 ~ 1.11)	0.10	0.71 (0.33 ~ 1.55)	0.39
Monthly income					Monthly income				
<1,000	1.00 (Reference)		1.00 (Reference)		<1,000	1.00 (Reference)		1.00 (Reference)	
1,000–2000	1.25 (0.71 ~ 2.19)	0.43	1.23 (0.69 ~ 2.20)	0.48	1,000–2000	0.74 (0.43 ~ 1.29)	0.29	0.90 (0.51 ~ 1.59)	0.71
2000–4,000	2.24 (1.40 ~ 3.60)	<0.01	1.86 (1.06 ~ 3.28)	0.03	2000–4,000	0.58 (0.36 ~ 0.94)	0.03	0.67 (0.38 ~ 1.18)	0.17
4,000–6,000	2.09 (1.33 ~ 3.29)	<0.01	1.67 (0.92 ~ 30.01)	0.09	4,000–6,000	0.69 (0.45 ~ 1.07)	0.10	0.68 (0.38 ~ 1.22)	0.20
≥6,000	2.76 (1.82 ~ 4.20)	<0.01	2.41 (1.32 ~ 4.40)	<0.01	≥6,000	0.80 (0.54 ~ 1.19)	0.27	0.70 (0.39 ~ 1.26)	0.24
Place of residence					Place of residence				
Urban areas	1.00 (Reference)		1.00 (Reference)		Urban areas	1.00 (Reference)		1.00 (Reference)	
Rural areas	0.59 (0.43 ~ 0.80)	<0.01	10.01 (0.70 ~ 1.45)	0.95	Rural areas	1.00 (0.73 ~ 1.36)	0.99	0.89 (0.62 ~ 1.29)	0.55
Basic social medical insurance					Basic social medical insurance				
Do not have	1.00 (Reference)		1.00 (Reference)		Do not have	1.00 (Reference)		1.00 (Reference)	
Have	3.21 (2.25 ~ 4.57)	<0.01	6.42 (2.27 ~ 18.16)	<0.01	Have	0.79 (0.58 ~ 1.10)	0.16	1.53 (0.51 ~ 4.57)	0.45
Commercial insurance					Commercial insurance				
Do not have	1.00 (Reference)		1.00 (Reference)		Do not have	1.00 (Reference)		1.00 (Reference)	
Have	1.09 (0.78 ~ 1.52)	0.61	1.13 (0.77 ~ 1.66)	0.54	Have	1.23 (0.88 ~ 1.73)	0.23	1.50 (10.01 ~ 2.24)	0.05
Out-of-pocket medical care					Out-of-pocket medical care				
Do not have	1.00 (Reference)		1.00 (Reference)		Do not have	1.00 (Reference)		1.00 (Reference)	
Have	0.28 (0.16 ~ 0.50)	<0.01	2.82 (0.88 ~ 9.06)	0.08	Have	1.32 (0.78 ~ 2.22)	0.31	1.91 (0.57 ~ 6.43)	0.29
Publicly-funded medical care					Publicly-funded medical care				
Do not have	1.00 (Reference)		1.00 (Reference)		Do not have	1.00 (Reference)		1.00 (Reference)	
Have	0.71 (0.43 ~ 1.17)	0.18	4.30 (1.37 ~ 13.49)	0.01	Have	1.52 (0.96 ~ 2.41)	0.08	2.46 (0.75 ~ 8.05)	0.14
Other medical security					Other medical security				
Do not have	1.00 (Reference)		1.00 (Reference)		Do not have	1.00 (Reference)		1.00 (Reference)	
Have	0.25 (0.12 ~ 0.49)	<0.01	1.72 (0.49 ~ 6.02)	0.39	Have	0.89 (0.44 ~ 1.78)	0.74	1.38 (0.37 ~ 5.14)	0.63
Education level					Education level				
Graduate student	1.00 (Reference)		1.00 (Reference)		Graduate student	1.00 (Reference)		1.00 (Reference)	
Bachelor	0.96 (0.71 ~ 1.30)	0.8	1.10 (0.79 ~ 1.54)	0.56	Bachelor	1.00 (0.73 ~ 1.38)	0.99	1.17 (0.83 ~ 1.65)	0.38
Junior college	0.93 (0.61 ~ 1.41)	0.73	1.25 (0.76 ~ 2.05)	0.39	Junior college	0.64 (0.40 ~ 1.02)	0.06	0.98 (0.56 ~ 1.69)	0.93
Technical secondary or high school	0.85 (0.54 ~ 1.36)	0.5	1.40 (0.77 ~ 2.53)	0.27	Technical secondary or high school	0.65 (0.39 ~ 1.08)	0.10	1.14 (0.61 ~ 2.14)	0.67
Middle school	0.50 (0.29 ~ 0.88)	0.02	0.97 (0.47 ~ 1.99)	0.94	Middle school	0.57 (0.31 ~ 1.05)	0.07	1.04 (0.48 ~ 2.23)	0.93
Primary school	0.14 (0.07 ~ 0.28)	<0.01	0.39 (0.16 ~ 0.94)	0.04	Primary school	1.24 (0.66 ~ 2.34)	0.50	1.96 (0.85 ~ 4.50)	0.11
Employment					Employment				
Currently employed	1.00 (Reference)		1.00 (Reference)		Currently employed	1.00 (Reference)		1.00 (Reference)	
Retired	0.71 (0.47 ~ 1.08)	0.11	1.52 (0.84 ~ 2.76)	0.17	Retired	0.60 (0.37 ~ 0.96)	0.03	0.69 (0.36 ~ 1.32)	0.26
Unemployed or jobless	0.46 (0.33 ~ 0.64)	<0.01	0.81 (0.50 ~ 1.33)	0.41	Unemployed or jobless	1.03 (0.75 ~ 1.43)	0.85	1.09 (0.66 ~ 1.82)	0.73
Occupation					Occupation				
Factory workers	1.00 (Reference)		1.00 (Reference)		Factory workers	1.00 (Reference)		1.00 (Reference)	
Company employees	1.10 (0.67 ~ 1.82)	0.7	1.07 (0.62 ~ 1.85)	0.80	Company employees	0.96 (0.55 ~ 1.67)	0.89	0.82 (0.46 ~ 1.47)	0.51
Government cadres	1.07 (0.54 ~ 2.11)	0.84	1.38 (0.67 ~ 2.83)	0.38	Government cadres	1.10 (0.53 ~ 2.27)	0.81	0.98 (0.46 ~ 2.11)	0.96
Healthcare workers	2.62 (1.64 ~ 4.18)	<0.01	2.86 (1.70 ~ 4.82)	<0.01	Healthcare workers	1.66 (1.01 ~ 2.73)	0.05	1.56 (0.90 ~ 2.69)	0.11
Teachers	1.15 (0.64 ~ 2.08)	0.65	1.34 (0.70 ~ 2.55)	0.38	Teachers	1.44 (0.78 ~ 2.66)	0.24	1.35 (0.69 ~ 2.63)	0.38
Business managers	0.76 (0.29 ~ 1.99)	0.57	1.08 (0.42 ~ 2.77)	0.87	Business managers	2.40 (1.06 ~ 5.47)	0.04	1.93 (0.82 ~ 4.53)	0.13
Freelancers	0.56 (0.30 ~ 1.07)	0.08	10.01 (0.51 ~ 1.99)	0.98	Freelancers	0.85 (0.42 ~ 1.72)	0.66	0.69 (0.33 ~ 1.46)	0.33
Students	0.96 (0.56 ~ 1.64)	0.87	3.03 (1.40 ~ 6.53)	<0.01	Students	1.59 (0.91 ~ 2.77)	0.10	0.94 (0.43 ~ 2.04)	0.87
Others	1.13 (0.66 ~ 1.94)	0.66	1.77 (0.98 ~ 3.18)	0.06	Others	0.79 (0.43 ~ 1.46)	0.46	0.68 (0.35 ~ 1.32)	0.25
Practice					Total				
Sex					Sex				
Male	1.00 (Reference)		1.00 (Reference)		Male	1.00 (Reference)		1.00 (Reference)	
Female	1.28 (1.00 ~ 1.63)	0.05	1.16 (0.88 ~ 1.53)	0.29	Female	1.22 (0.93 ~ 1.61)	0.16	1.06 (0.79 ~ 1.42)	0.70
Age					Age				
19–34	1.00 (Reference)		1.00 (Reference)		19–34	1.00 (Reference)		1.00 (Reference)	
35–49	0.73 (0.56 ~ 0.96)	0.03	0.97 (0.69 ~ 1.37)	0.88	35–49	1.18 (0.88 ~ 1.58)	0.27	1.30 (0.92 ~ 1.86)	0.14
50–64	0.32 (0.22 ~ 0.46)	<0.01	0.78 (0.49 ~ 1.26)	0.31	50–64	0.39 (0.26 ~ 0.60)	<0.01	0.73 (0.44 ~ 1.24)	0.24
≥65	0.12 (0.07 ~ 0.22)	<0.01	0.29 (0.13 ~ 0.60)	<0.01	≥65	0.15 (0.08 ~ 0.28)	<0.01	0.27 (0.12 ~ 0.61)	<0.01
Monthly income					Monthly income				
<1,000	1.00 (Reference)		1.00 (Reference)		<1,000	1.00 (Reference)		1.00 (Reference)	
1,000–2000	0.76 (0.45 ~ 1.28)	0.3	0.84 (0.48 ~ 1.49)	0.55	1,000–2000	1.05 (0.58 ~ 1.93)	0.87	1.21 (0.64 ~ 2.29)	0.56
2000–4,000	0.93 (0.61 ~ 1.44)	0.75	1.12 (0.64 ~ 1.94)	0.70	2000–4,000	1.41 (0.85 ~ 2.33)	0.18	1.25 (0.68 ~ 2.30)	0.48
4,000–6,000	1.23 (0.82 ~ 1.86)	0.31	1.13 (0.64 ~ 2.02)	0.67	4,000–6,000	1.84 (1.14 ~ 2.98)	0.01	1.10 (0.58 ~ 2.08)	0.77
≥6,000	1.80 (1.24 ~ 2.63)	<0.01	1.41 (0.78 ~ 2.54)	0.26	≥6,000	2.86 (1.84 ~ 4.45)	<0.01	1.51 (0.79 ~ 2.87)	0.21
Place of residence					Place of residence				
Urban areas	1.00 (Reference)		1.00 (Reference)		Urban areas	1.00 (Reference)		1.00 (Reference)	
Rural areas	0.55 (0.41 ~ 0.73)	<0.01	0.82 (0.57 ~ 1.18)	0.28	Rural areas	0.48 (0.35 ~ 0.67)	<0.01	0.89 (0.60 ~ 1.32)	0.56
Basic social medical insurance					Basic social medical insurance				
Do not have	1.00 (Reference)		1.00 (Reference)		Do not have	1.00 (Reference)		1.00 (Reference)	
Have	3.34 (2.40 ~ 4.66)	<0.01	10.05 (3.47 ~ 29.09)	<0.01	Have	3.97 (2.69 ~ 5.88)	<0.01	17.48 (5.94 ~ 51.41)	<0.01
Commercial insurance					Commercial insurance				
Do not have	1.00 (Reference)		1.00 (Reference)		Do not have	1.00 (Reference)		1.00 (Reference)	
Have	1.03 (0.75 ~ 1.42)	0.85	1.04 (0.70 ~ 1.53)	0.86	Have	1.38 (0.98 ~ 1.95)	0.07	1.47 (0.97 ~ 2.21)	0.07
Out-of-pocket medical care					Out-of-pocket medical care				
Do not have	1.00 (Reference)		1.00 (Reference)		Do not have	1.00 (Reference)		1.00 (Reference)	
Have	0.22 (0.13 ~ 0.38)	<0.01	3.47 (1.07 ~ 11.32)	0.04	Have	0.23 (0.13 ~ 0.42)	<0.01	7.44 (2.19 ~ 25.29)	<0.01
Publicly-funded medical care					Publicly-funded medical care				
Do not have	1.00 (Reference)		1.00 (Reference)		Do not have	1.00 (Reference)		1.00 (Reference)	
Have	0.63 (0.39 ~ 1.02)	0.06	6.96 (2.20 ~ 22.09)	<0.01	Have	0.67 (0.39 ~ 1.15)	0.14	11.92 (3.62 ~ 39.24)	<0.01
Other medical security					Other medical security				
Do not have	1.00 (Reference)		1.00 (Reference)		Do not have	1.00 (Reference)		1.00 (Reference)	
Have	0.49 (0.26 ~ 0.93)	0.03	4.35 (1.24 ~ 15.22)	0.02	Have	0.30 (0.15 ~ 0.62)	<0.01	5.54 (1.48 ~ 20.73)	0.01
Education level					Education level				
Graduate student	1.00 (Reference)		1.00 (Reference)		Graduate student	1.00 (Reference)		1.00 (Reference)	
Bachelor	0.76 (0.56 ~ 1.03)	0.08	0.77 (0.54 ~ 1.09)	0.14	Bachelor	0.84 (0.61 ~ 1.16)	0.29	1.03 (0.72 ~ 1.48)	0.86
Junior college	0.35 (0.23 ~ 0.53)	<0.01	0.50 (0.30 ~ 0.82)	<0.01	Junior college	0.47 (0.29 ~ 0.75)	<0.01	0.76 (0.44 ~ 1.31)	0.32
Technical secondary or high school	0.24 (0.15 ~ 0.38)	<0.01	0.44 (0.24 ~ 0.80)	<0.01	Technical secondary or high school	0.28 (0.16 ~ 0.47)	<0.01	0.67 (0.34 ~ 1.30)	0.23
Middle school	0.09 (0.05 ~ 0.17)	<0.01	0.20 (0.10 ~ 0.42)	<0.01	Middle school	0.14 (0.08 ~ 0.26)	<0.01	0.36 (0.16 ~ 0.81)	0.01
Primary school	0.03 (0.02 ~ 0.07)	<0.01	0.10 (0.04 ~ 0.24)	<0.01	Primary school	0.05 (0.02 ~ 0.10)	<0.01	0.16 (0.06 ~ 0.39)	<0.01
Employment					Employment				
Currently employed	1.00 (Reference)		1.00 (Reference)		Currently employed	1.00 (Reference)		1.00 (Reference)	
Retired	0.29 (0.19 ~ 0.43)	<0.01	1.45 (0.81 ~ 2.62)	0.21	Retired	0.26 (0.16 ~ 0.41)	<0.01	1.36 (0.70 ~ 2.63)	0.36
Unemployed or jobless	0.54 (0.40 ~ 0.73)	<0.01	0.84 (0.51 ~ 1.37)	0.48	Unemployed or jobless	0.36 (0.25 ~ 0.51)	<0.01	0.82 (0.47 ~ 1.41)	0.47
Occupation					Occupation				
Factory workers	1.00 (Reference)		1.00 (Reference)		Factory workers	1.00 (Reference)		1.00 (Reference)	
Company employees	1.33 (0.83 ~ 2.12)	0.23	0.97 (0.58 ~ 1.62)	0.90	Company employees	1.30 (0.75 ~ 2.25)	0.35	0.95 (0.52 ~ 1.74)	0.87
Government cadres	0.79 (0.41 ~ 1.52)	0.48	0.73 (0.36 ~ 1.45)	0.37	Government cadres	1.06 (0.49 ~ 2.27)	0.88	1.13 (0.51 ~ 2.52)	0.76
Healthcare workers	5.39 (3.40 ~ 8.55)	<0.01	3.62 (2.17 ~ 6.04)	<0.01	Healthcare workers	4.60 (2.78 ~ 7.63)	<0.01	3.67 (2.08 ~ 6.48)	<0.01
Teachers	1.03 (0.59 ~ 1.80)	0.93	0.66 (0.35 ~ 1.24)	0.20	Teachers	1.58 (0.83 ~ 30.01)	0.17	1.30 (0.64 ~ 2.66)	0.46
Business managers	0.89 (0.36 ~ 2.17)	0.79	1.07 (0.43 ~ 2.62)	0.89	Business managers	1.52 (0.54 ~ 4.23)	0.43	1.83 (0.68 ~ 4.89)	0.23
Freelancers	0.60 (0.32 ~ 1.10)	0.1	1.12 (0.58 ~ 2.16)	0.73	Freelancers	0.66 (0.33 ~ 1.32)	0.24	1.21 (0.58 ~ 2.54)	0.61
Students	2.90 (1.75 ~ 4.83)	<0.01	3.63 (1.71 ~ 7.70)	<0.01	Students	1.60 (0.89 ~ 2.85)	0.11	2.28 (0.98 ~ 5.29)	0.06
Others	0.86 (0.52 ~ 1.42)	0.56	1.51 (0.86 ~ 2.67)	0.16	Others	0.90 (0.50 ~ 1.64)	0.74	1.51 (0.78 ~ 2.91)	0.22

OR, odds ratio; CI, confidence interval.

### 3.3 Construction of ML-based predictive models

**Figure 1:**
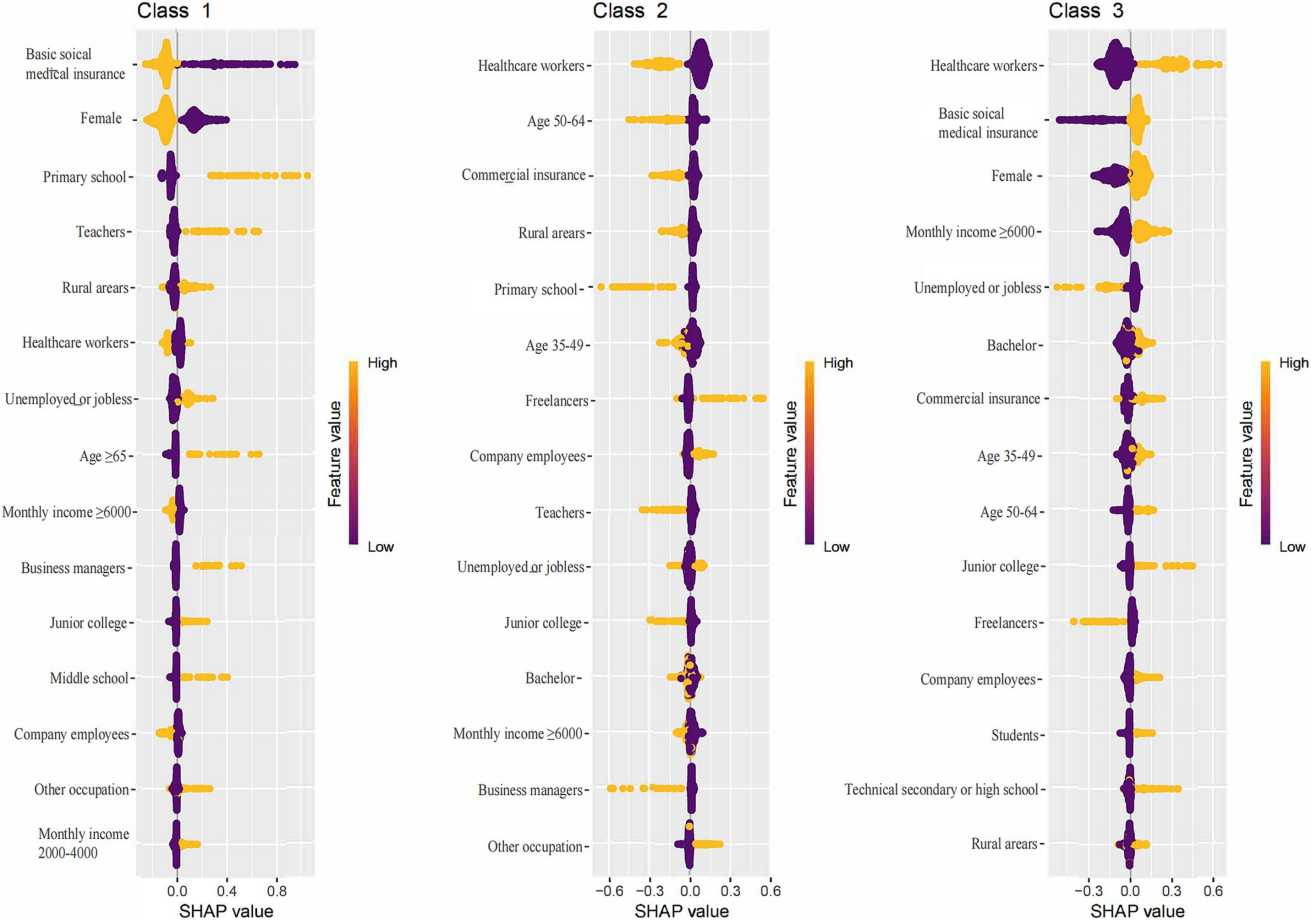
Feature importance ranked by the mean absolute magnitude of SHAP values within the XGB model for knowledge prediction. The SHAP values are a measure of a feature’s contribution to the prediction, with positive values indicating an increase in the prediction and negative values indicating a decrease. SHAP: SHapley Additive exPlanation; XGB: eXtreme Gradient Boosting; Class 1: poor; Class 2: adequate; Class 3: excellent.

**Figure 2:**
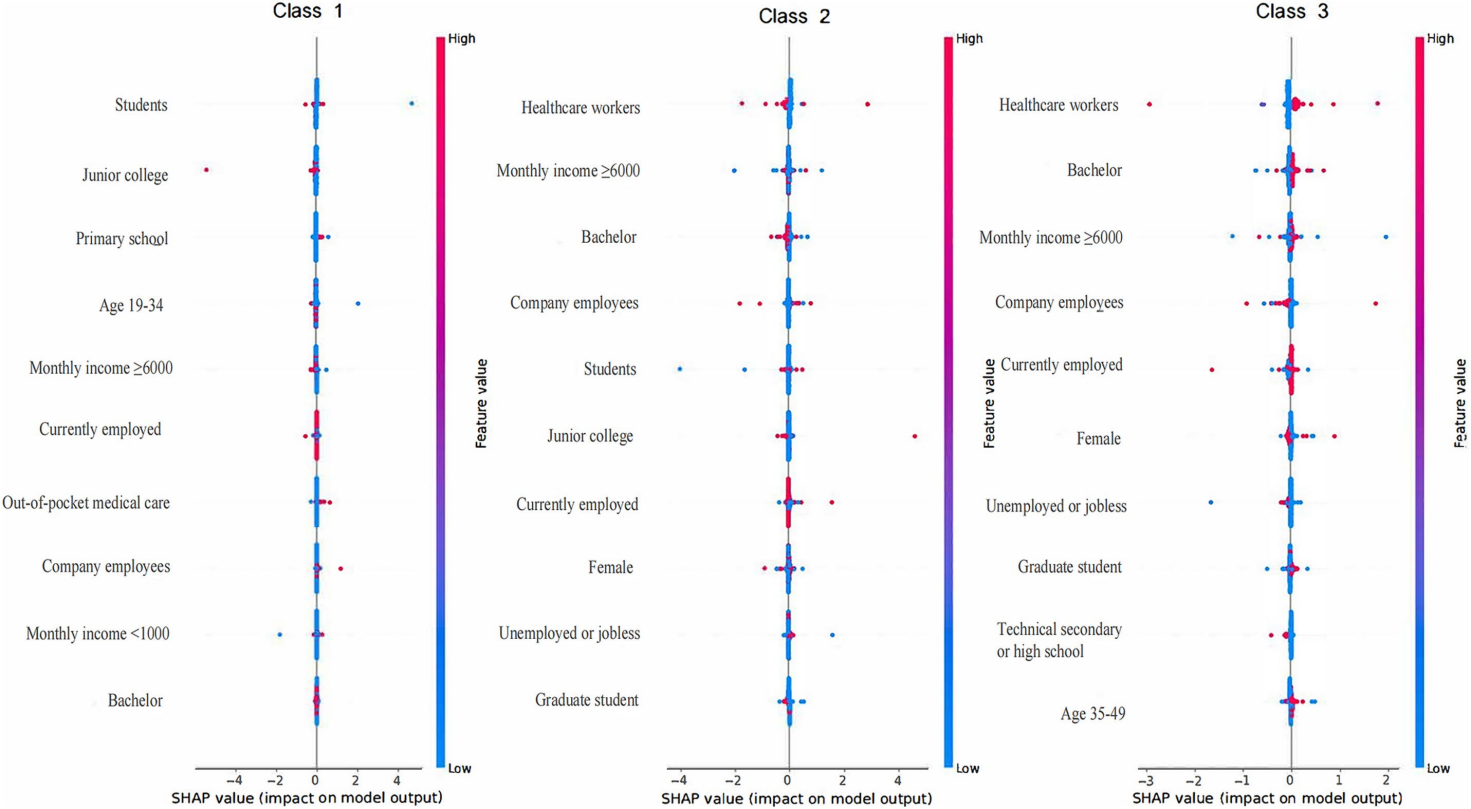
Feature importance (top 10 features) ranked by the mean absolute magnitude of SHAP values within the FCNN model for attitude prediction. The SHAP values are a measure of a feature’s contribution to the prediction, with positive values indicating an increase in the prediction and negative values indicating a decrease. SHAP: SHapley Additive exPlanation; FCNN: Fully Connected Neural Network model; Class 1: poor; Class 2: adequate; Class 3: excellent.

**Figure 3:**
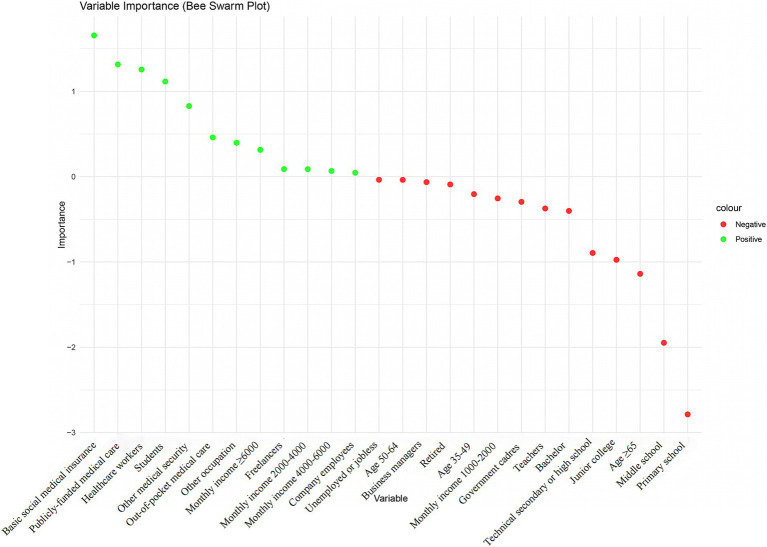
Bee-swarm plots of feature importance within the Ordered Multinomial Logistic Regression model for practice prediction. The *x*-axis represents the individual variables and the y-axis indicates their importance scores. Variables are color-coded to denote their impact on the model’s output: green for positive influence and red for negative influence. Class 1: poor; Class 2: adequate; Class 3: excellent.

**Figure 4:**
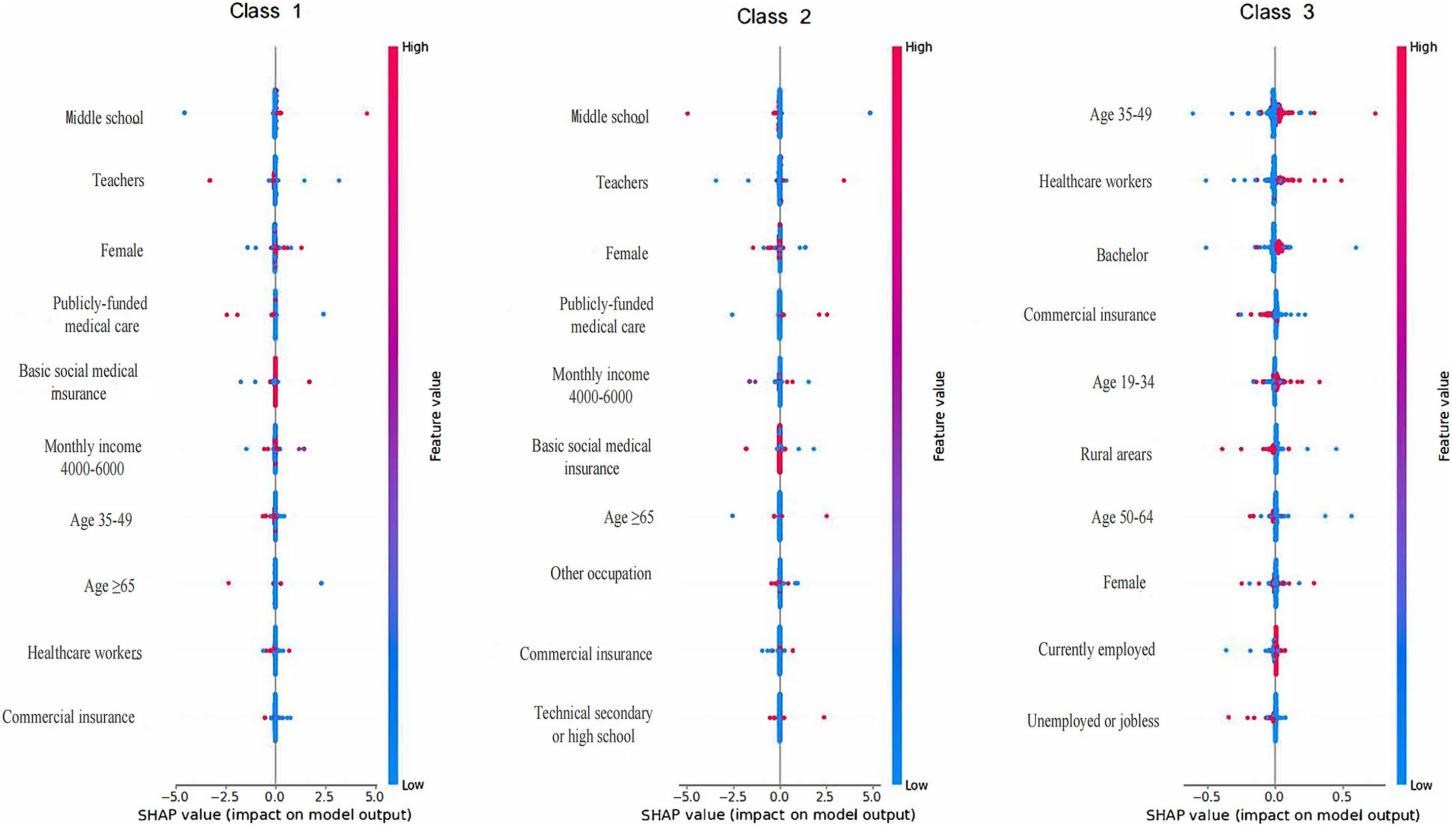
Feature importance (top 10 features) ranked by the mean absolute magnitude of SHAP values within the DNN model for total score prediction. The SHAP values are a measure of a feature’s contribution to the prediction, with positive values indicating an increase in the prediction and negative values indicating a decrease. SHAP: SHapley Additive exPlanation; DNN: Deep Neural Network; Class 1: poor; Class 2: adequate; Class 3: excellent.

ML techniques were employed to construct and select the most optimal predictive models for KAP and total scores. For knowledge prediction, the XGB model demonstrated the best predictive performance (training set accuracy: 0.7014, Kappa: 0.3045; validation set accuracy: 0.6186, Kappa: 0.1004), with variable importance displayed in Figure 1. The FCNN model was selected for attitude prediction (training set accuracy: 0.7205, Kappa: 0.0778; validation set accuracy: 0.7019, Kappa: 0.0008), with variable importance illustrated in Figure 2. The Ordered Multinomial Logistic Regression model was chosen for its superior predictive performance in the practice model (training set accuracy: 0.6471, Kappa: 0.3421; validation set accuracy: 0.6302, Kappa: 0.3153), with variable importance shown in Figure 3. Lastly, the DNN model showed the best prediction effect on the total score (training set accuracy: 0.7387, Kappa: 0.3211; validation set accuracy: 0.7074, Kappa: 0.1902), with variable importance presented in Figure 4. The performance metrics of precision, recall, and F1 score for the four models are depicted in Table 3.

**Table 3:** Machine learning analysis results of these four selected models (training set and validation set results).

	Precision	Recall	F1 score
Knowledge model			
Training set			
Class 1	95.24%	37.04%	53.33%
Class 2	68.96%	94.73%	79.81%
Class 3	72.62%	27.60%	40.00%
Validation set			
Class 1	33.33%	8.70%	13.79%
Class 2	64.31%	88.72%	74.57%
Class 3	48.65%	19.15%	27.48%
Attitude model			
Training set			
Class 1	71.90%	99.80%	83.60%
Class 2	78.60%	7.90%	14.40%
Class 3	0.00%	0.00%	0.00%
Validation set			
Class 1	70.80%	98.60%	82.40%
Class 2	25.00%	1.70%	3.20%
Class 3	0.00%	0.00%	0.00%
Practice model			
Training set			
Class 1	72.73%	17.78%	28.57%
Class 2	62.18%	68.97%	65.40%
Class 3	67.37%	66.57%	66.96%
Validation set			
Class 1	55.56%	27.78%	37.04%
Class 2	60.12%	67.79%	63.72%
Class 3	67.16%	62.50%	64.75%
Total score model			
Training set			
Class 1	90.30%	53.80%	67.50%
Class 2	73.90%	96.90%	83.80%
Class 3	56.70%	10.10%	17.20%
Validation set			
Class 1	42.90%	14.30%	21.40%
Class 2	72.00%	95.40%	82.10%
Class 3	60.00%	12.50%	20.70%

Knowledge model: eXtreme Gradient Boosting model; Attitude model: Fully Connected Neural Network model; Practice model: the Ordered Multinomial Logistic Regression model; Total score model: Deep Neural Network model; Class 1: poor; Class 2: adequate; Class 3: excellent.

## 4 Discussion

This study conducted an in-depth analysis of the current state of medication KAP among permanent residents of Hubei Province through a questionnaire survey. Developing precise prediction models necessitates the integration of diverse and sophisticated artificial intelligence algorithms. Prior research endeavors to predict medication risk among residents based on a KAP survey predominantly utilized traditional bio-statistical multivariate logistic regression methods (18–25). In contrast, our study incorporates seven algorithms, encompassing traditional LR alongside six advanced ML techniques from the realm of AI. By rigorously comparing the predictive capabilities of models formulated through these various algorithms, we have identified the model that exhibits the optimal predictive performance. Furthermore, previous similar research has improperly categorized outcome indicators as binary variables. In our study, however, we analyze these outcome indicators as ordinal scaled variables to achieve a more accurate prediction model (26). We utilized the collected data to employ multivariate logistic regression analysis and ML techniques to establish predictive models. The aim was to identify key factors influencing residents’ medication behaviors and to provide a scientific basis for developing targeted intervention measures.

The research revealed significant disparities in medication KAP among Hubei Province residents. Our study found that 46.3% of residents in Hubei Province achieved excellence in medication practice, while only 10.3% attained excellence in medication attitude and 30.2% in medication knowledge. When compared to other regions, such as Huizhou (4.6% excellence in attitude, 64.5% in knowledge, and 55.0% in practice) and Wuhan (6.9% excellence in attitude, 51.9% in knowledge, and 38.3% in practice) (27, 28), it is evident that there are variations in medication KAP across different regions. This initiative emphasizes that systemic gaps in medication safety education and healthcare infrastructure, especially in low-and middle-income regions, can lead to discrepancies in medication knowledge and attitudes among residents. Regarding knowledge, while many residents have a basic understanding of medication, a significant portion still lacks comprehensive insight into drug information, usage precautions, and potential adverse effects. This knowledge gap may lead to erroneous self-medication decisions, increasing medication risks. In terms of attitudes, although residents generally recognize the importance of medication safety, practical constraints such as financial pressures and medication inconvenience may lead some to independently alter their medication regimens, compromising treatment efficacy. At the practical level, medication adherence varies widely, with some residents failing to maintain standardized medication due to insufficient supervision or guidance, leading to recurrent or exacerbated illnesses.

Through univariate and multivariate analyses, we identified several key factors-including age, monthly income, education level, occupation, residence location, basic medical insurance, and medical payment methods-that significantly influence residents’ medication KAP scores and overall performance. Notably, younger individuals, those with higher incomes, higher educational attainment, urban residents, and those with comprehensive medical insurance showed better performance in medication KAP. Our result was consistent with previous study conducted among residents in Harbin and Shanxi, China (26, 29). This could be due to their greater access to accurate medication information, increased health investment focus, and superior medical resources. The internet has become a pivotal source for accessing information related to medicines. Individuals who possess a heightened ability to navigate the internet effectively are better positioned to efficiently gather medicine - related information. A study examining the use of the internet for searching for information on medicines and disease among the Italian population revealed that gender, age, social status, and educational level significantly influence searching behaviors and usage patterns (30). Younger individuals and those with higher educational attainment are more efficient at acquiring medication knowledge and practices. In contrast, those with lower levels of education tend to acquire their knowledge of medication use via personal experiences and instinctive approaches, and they exhibit a less developed perception of the risks linked to medication mistakes (31). Occupational disparities also significantly affect medication behaviors, likely due to variations in working environments, health awareness, and economic circumstances among different occupational groups, which was similar with a study conducted in Egypt (32). In the univariate analysis, women demonstrated significantly higher medication knowledge and practice scores than men, which was consistent with previous studies (26, 32). However, in the multivariate regression analysis, the statistical significance of gender on medication knowledge became non-significant after adjusting for other variables. This indicates that gender differences in medication knowledge and practice may be confounded by other factors like residence, education level, and employment status. Gender differences in knowledge scores may reflect sociocultural roles where women often assume primary responsibility for family health management. While females may engage more actively in health information seeking, practical medication behaviors are moderated by socioeconomic factors.

We also employed ML techniques to establish predictive models for medication KAP and overall scores. The findings indicated that the Ordered Multinomial Logistic Regression model was best for predicting knowledge, the XGB model was superior for behavior prediction, the FCNN model performed well for attitude prediction, and the DNN model showed the best results for predicting the overall score. The clinical and public health utility of our ML models is demonstrated through their ability to enable precision interventions and inform policy-making. The XGBoost model excels in identifying high-risk subgroups (e.g., rural older adult with low education levels) for targeted interventions, such as simplified medication guides and community pharmacist outreach programs. The FCNN model provides real-time monitoring of medication safety attitudes via mobile-integrated dynamic questionnaires, enabling rapid adaptation of educational campaigns during public health events (e.g., post-vaccine rollout hesitancy), akin to ECG-based cardiac care systems. The DNN model synthesizes multi-dimensional predictors to guide policy, such as expanding insurance coverage for medication counseling among “high-knowledge, low-practice” urban professionals.

Based on the analysis, we propose the following recommendations to improve medication safety for Hubei Province residents: (1) Strengthen pharmaceutical education dissemination, particularly among the older adult, rural areas, and low-income populations. By integrating online and offline methods, we should provide accessible and user-friendly pharmaceutical education resources to enhance their self-medication capabilities. (2) Optimize the healthcare service system and foster better doctor-patient communication to ensure patients fully understand and adhere to medical instructions. Medical institutions should establish dedicated pharmacist positions to offer personalized and professional medication counseling. Additionally, refine the medical insurance system to alleviate the financial burden of medication for residents, improving medication accessibility and affordability. Strengthen oversight of the pharmaceutical market, combat illegal drug sales, regulate pharmaceutical advertisements, and curb the spread of misleading information. (3) Utilize advanced information technology to develop intelligent medication management systems that can assist residents in tracking their medication history, reminding them of medication times, monitoring drug interactions, and ultimately enhancing medication adherence and safety. (4) Promote scientific research collaboration and exchanges to explore effective strategies for improving medication safety among residents, including conducting research projects related to medication safety and establishing comprehensive medication safety monitoring systems.

This study has several limitations. First, the cross-sectional design restricts causal inference and temporal interpretation of variable relationships. Second, underrepresentation of older adult populations (≥65 years: 5.47%) and rural residents (22.94%) may limit generalizability. Third, while 10-fold cross-validation and BORUTA variable selection mitigated overfitting, external validation across diverse regions remains pending. Future work will leverage the Chinese Pharmaceutical Association’s CMEI database for cross-province validation. Additionally, online data collection may exclude digitally underserved populations, introducing selection bias. To enhance ecological validity, future studies should integrate electronic health records (EHRs) and community surveillance data using mixed-methods designs.

## 5 Conclusion

This study reveals critical disparities in medication safety practices among residents of Hubei Province. Our ML framework-specifically the XGBoost model for knowledge prediction and DNN for total score synthesis-proved effective in identifying high-risk subgroups, particularly rural older adult individuals with low education levels. While gender disparities in knowledge acquisition were statistically significant, practical behaviors were predominantly influenced by systemic factors, including inadequate medical insurance coverage and rural healthcare resource gaps. Future interventions should integrate AI-driven risk stratification with community pharmacist programs, particularly targeting populations with limited health literacy. These findings align with the WHO’s *Medication Without Harm* initiative by highlighting actionable pathways for precision interventions.

## Data Availability

The raw data supporting the conclusions of this article will be made available by the authors, without undue reservation.

## Footnotes

^1^ https://www.wjx.cn/wjx/design/previewmobile.aspx?activity=263702888&s=1
